# Long-term HIV-1 Tat Expression in the Brain Led to Neurobehavioral, Pathological, and Epigenetic Changes Reminiscent of Accelerated Aging

**DOI:** 10.14336/AD.2019.0323

**Published:** 2020-02-01

**Authors:** Xiaojie Zhao, Yan Fan, Philip H. Vann, Jessica M. Wong, Nathalie Sumien, Johnny J. He

**Affiliations:** ^1^Department of Microbiology, Immunology & Genetics and; ^2^Department of Pharmacology & Neuroscience, Graduate School of Biomedical Sciences, University of North Texas Health Science Center, Fort Worth, Texas 76107, USA

**Keywords:** HIV-1 Tat, accelerated aging, DNA methyltransferases, DNA methylation

## Abstract

HIV infects the central nervous system and causes HIV/neuroAIDS, which is predominantly manifested in the form of mild cognitive and motor disorder in the era of combination antiretroviral therapy. HIV Tat protein is known to be a major pathogenic factor for HIV/neuroAIDS through a myriad of direct and indirect mechanisms. However, most, if not all of studies involve short-time exposure of recombinant Tat protein *in vitro* or short-term Tat expression *in vivo*. In this study, we took advantage of the doxycycline-inducible brain-specific HIV-1 Tat transgenic mouse model, fed the animals for 12 months, and assessed behavioral, pathological, and epigenetic changes in these mice. Long-term Tat expression led to poorer short-and long-term memory, lower locomotor activity and impaired coordination and balance ability, increased astrocyte activation and compromised neuronal integrity, and decreased global genomic DNA methylation. There were sex- and brain region-dependent differences in behaviors, pathologies, and epigenetic changes resulting from long-term Tat expression. All these changes are reminiscent of accelerated aging, raising the possibility that HIV Tat contributes, at least in part, to HIV infection-associated accelerated aging in HIV-infected individuals. These findings also suggest another utility of this model for HIV infection-associated accelerated aging studies.

HIV-1 infection of the central nervous system (CNS) often causes neurological symptoms that include motor and cognitive dysfunction [1], which are collectively called neuroAIDS. In the era of combination anti-retroviral therapy (cART), a more discrete form of CNS dysfunction so-called minor cognitive motor disorder (MCMD) has become more common [2-4]. At the cellular level, the primary cell targets for HIV infection are macrophages/microglia and, to a lesser extent, astrocytes [5-10]. Neurons that are mostly affected in the brain of HIV-infected individuals are rarely infected. Therefore, a number of indirect mechanisms have been proposed for HIV/neuroAID pathogenesis.

Among these indirect mechanisms is HIV-1 Tat protein, a major pathogenic factor in HIV/neuroAIDS. Tat protein is detected in the brain of HIV-infected individuals without cART [11] and with cART [12]. Tat is secreted from HIV-infected-microglia/macrophages and astrocytes [13, 14]. Neurons or other brain cells can take up the extracellular Tat protein [15-17]. Tat can adversely affect neurons in both direct and indirect manners. Direct exposure of Tat alters neuronal integrity, homeostasis, neuroexcitatory property, endoplasmic reticulum (ER) calcium load, and oxidative state [18-23]. Tat can also affect neuron survival indirectly by recruiting monocytes/macrophages and lymphocytes into the CNS [24-29], or by altering neuronal gene expression profiles and intracellular signaling cascades [17, 30, 31]. In agreement with these findings, Tat expression in or injection into the CNS in the absence of HIV-1 infection is sufficient to cause neuropathologies similar to most of those noted in the brain of HIV-infected individuals [29, 32]. In addition, Tat interaction with other brain cells, astrocytes in particular, also plays a significant role in Tat neurotoxicity and HIV/neuroAIDS [33-45]. Tat alters astrocytes and neurons to form aggregates and the neurite processes to coalesce into fascicles in cultures [46]. Tat also induces expression of several other cytokines and chemokines in astrocytes, including IL-1β, IL-6, Rantes, and CXCL10 [47-50]. We have shown that Tat activates glial fibrillary acidic protein (GFAP) expression in astrocytes and subsequently impairs astrocyte function and results in neuron death [35, 51, 52]. In addition, we have shown that the transcriptional co-activator p300 regulates Tat-induced GFAP up-regulation through transcription factor early growth response 1 and p300 [52-54]. Furthermore, using the doxycycline-inducible brain-specific HIV-1 Tat transgenic mouse model, we and others have shown that Tat alters autophagy, ER stress, lysosomal exocytosis, neurite growth, and neurogenesis [55-59]. Besides these changes, Tat has been shown to induce abnormal behaviors in mice and rats, such as learning and memory deficits, sensorimotor impairment, anxiety, and depressive-like behavior [60-69], which are accompanied by neuropathological changes such as astrocytosis and compromised neuronal integrity [32, 52, 62, 67]. It is important to note that most if not all these studies involved the use of short-term exposure of recombinant Tat protein *in vitro* or short-term Tat expression *in vivo*. Thus, the effects of long-term Tat expression are not known.

In the study, we aimed to determine the effects of long-term Tat expression in the brain on behaviors, pathology, and epigenetic landscape. We took advantage of the doxycycline (Dox)-inducible brain-specific HIV-1 Tat transgenic mouse model (iTat) [70] and fed the animals with Dox-containing diet for 12 months (equivalent to people living with HIV infection for 50 years). Then, we performed a series of behavioral tests, analyzed astrocyte activation and neuronal integrity by assessing GFAP, synaptophysin (SYP), and post-synaptic density protein 95 (PSD 95) expression in the brain, and performed genomic DNA methylation analysis.

## MATERIALS AND METHODS

### Animals

Dox-inducible and astrocyte-specific HIV-1 Tat-transgenic mice (iTat) were generated as previously described [32]. Both wild-type (C57BL/6) and iTat mice at postnatal day 21 were fed with DOX-containing diet (0.625g/kg, Envigo, Indianapolis, IN) for 12 months, and then were subjected to behavioral tests using a computerized video tracking system (Anymaze, Stoelting Co., Wood Dale, IL). At the end of the behavioral tests, mice were euthanized, and the brains were collected. All the animal procedures were approved by the Institutional Animal Care and Use Committee. Mice were housed with a 12-hour light and 12-hour dark photoperiod and provided water and food *ad libitum*.

### Behavioral tests

**Figure 1.**
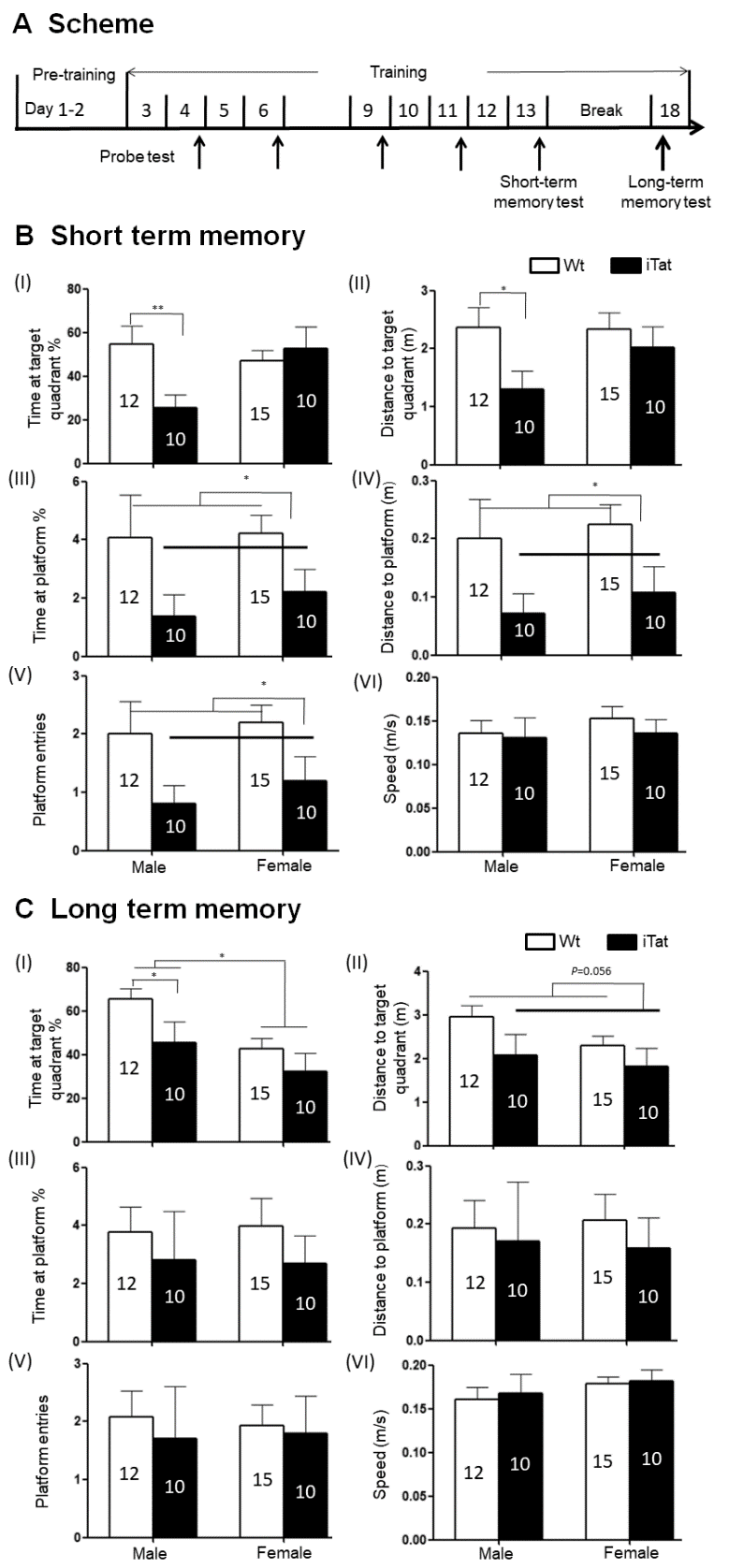
**Spatial memory of iTat mice by Morris water maze (MWZ)**. Wild-type (Wt) and iTat mice of 21 days old were fed with Dox-containing food pellets for 12 months and their short- and long-term spatial memory were determined. **(A)** Scheme of MWZ test. Mice underwent pre-training for 2 days, then training every day and probe test (marked by thin arrows) every other day, and the data in last probe test (Day 13) was analyzed to determine short-term spatial memory. After 7 days, the mice underwent another probe test (marked by a thick arrow) on day 18 to determine long-term spatial memory. (B and C) iTat short-term spatial memory (B) and long-term spatial memory (C). Mice were grouped into males and females and assessed for spatial memory based on the Time at target quadrant % (I), Distance to target quadrant (II), Time at platform site % (III), Distance in platform site (IV), Platform entries (V), and Speed (VI). The number of mice in each group was shown in the bar.

Three behavioral tests were performed: Morris water maze, open field test, and bridge walking test to determine spatial memory, spontaneous locomotor activity, and balance, respectively. Morris water maze was performed using a slightly modified protocol [71] (Fig. 1A). The test consisted of two stages (pre-training and acquisition/probe) with a hidden platform (1.5 cm below surface) in 24±1^o^C, opaque water. The pre-training phase is set so that the mice can learn to swim and climb onto the hidden platform. During pre-training, the mice were given a maximum of 60s to reach the platform at the end of a straight alley. If the mice failed to reach to the platform within 60s, they would be directed towards the platform and allowed to stay on the platform for 15s. Each mouse had two pre-training sessions, one on day 1 and one on day 2. There were five trials in each pre-training session with a 2-min interval between each trial. During the first pre-training stage, a black curtain was used to cover the tank to hide surrounding visual cues. The second stage comprised an acquisition phase followed by a delayed probe test. All trials at this stage were conducted in the tank with no covering curtain, and the mice were expected to use the surrounding visual cues in the room for navigation. For each trial, mice were randomly put into one quadrant and held facing to the tank wall before they were released to swim. The maximum time allowed for mice to swim to reach the platform was 90s and to stay on the platform was 10s. If the mice failed to reach to the platform within 90s, they would be directed towards the platform and allowed to stay on the platform for 15s. There were a total of nine training sessions, one every day (Day 3-13, except for the weekends), comprising five trials with 2-min intervals between each trial. In addition to the training, probe trials (marked by arrows) were conducted on Day 4, 6, 9, 11, and 13 as the 5^th^ trials and lasted 30 seconds. During the probe trial, the platform was removed to prevent the mice from climbing onto it. The probe trial sessions on these days were used to determine the short-term memory. After a 7-day break, the 10^th^ session that only consisted of one probe trial was conducted to determine the long-term memory. For open field test, each mouse was placed into a clear acrylic chamber (40.5×40.5×30.5cm) and allowed to freely move around the chamber for 10 min. Travel distance and speed were recorded using the AnyMaze software. For bridge walking test, four bridge beams with two sizes (small and large) and two shapes (round and square) were used to assess the balance ability at four levels of difficulties. The test order from easy to hard in difficulties was large square (LS), large round (LR), small square (SS) and small round (SR). The latency to fall or reach a safe platform was record after mice were placed on the beam, and the maximum time was set for 60s. Each bridge was tested three times and the average latency to fall was calculated.

### 5-methylcytosine ELISA

Genomic DNA was extracted from mouse brain tissues or cultured cells using a DNA extraction kit (Promega, Madison, WI). The quantity and quality of genomic DNA were determined by Nanodrop (ThermoFisher, Waltham, MA). One hundred nanograms of genomic DNA were used to determine the 5-methylcytosine level using an ELISA kit (Zymo, Irvine, CA) according to the manufacturer’s instructions.

### Western blotting

Brain tissues or cells were lysed in RIPA buffer containing protease inhibitor mixture (Roche, Indianapolis, IN) and briefly sonicated on ice. Protein concentration was determined using a Bio-Rad DC protein assay kit (Bio-Rad, Hercules, CA). Lysates were electrophoretically separated by 8% SDS-polyacrylamide gel and blotted, and probed with appropriate primary antibodies: DNMT3B (1:200, Santa Cruz Biotechnologies, Santa Cruz, CA), PSD95 (1:1000, Abcam, Cambridge, MA), SYP (1:500, Santa Cruz Biotechnologies), GFAP (1:1000, DAKO, Santa Clara, CA) and β-actin (1:2000, Sigma-Aldrich, St. Louis, MO). Proteins levels on the blots were quantitated using a Bio-Rad ChemicDoc imaging system (Bio-Rad, Hercules, CA).

### Real-Time reverse transcription PCR (qRT-PCR)

Total RNA was isolated from cells using TRIzol (Life Technology, Carlsbad, CA). cDNA was synthesized from 1 μg RNA using a Script II RT kit (Promega) and used as a template for qPCR using a SYBR Green kit (Bio-Rad). Bio-Rad CFX Manager Software was used to calculate gross-threshold (CT) values. The 2^(-△△CT)^ was calculated to represent the fold change of gene expression and normalized using β-actin as a reference. All the primers was used as follows: DNMT1: forward: 5’-CTT CAC CTA GTT CCG TGG CTA-3’, reverse: 5’-CCC TCT TCC GAC TCT TCC TT-3’; DNMT3A: forward: 5’-TCC ATG AAA ATG GAG GGC TC-3’, reverse: 5’-TTG CTG ATG TAG TAG GGG TC-3’; DNMT3B: forward: 5’-GAT GAG GAG AGC CGA GAA CG-3’, reverse: 5’-CAG AGC CCA CCC TCA AAG AG-3’; β-actin: forward: 5’-AGA GAA GTG GGG TGG CTT TT -3’, reverse: 5’-AAA CTG GAA CGG TGA AGG TG -3’.

### Data analysis

Unless stated otherwise, all the data were analyzed by two-way ANOVA and Fisher's Least Significant Difference (LSD) for post hoc tests. *p*<0.05 was considered significant and marked as *; *p*<0.01 and *p*<0.001 were both considered highly significant and marked as ** and ***, respectively.

**Figure 2.**
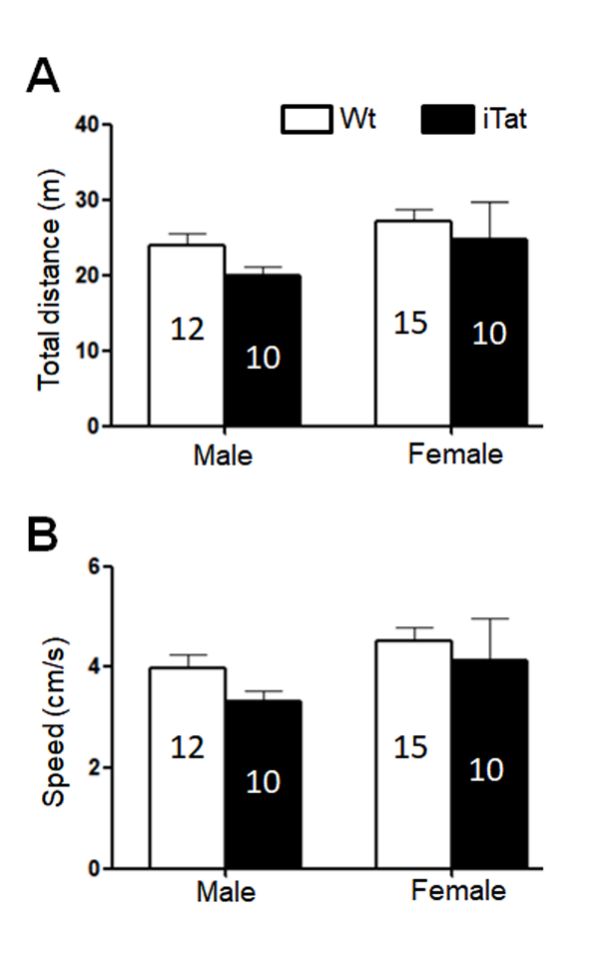
**Locomotor activity of iTat mice by open field test**. The mice were placed into an open chamber and their movement was recorded for 10 min. The trace was analyzed for total travel distance (A) and travel speed (B). The number of mice in each group was shown in the bar.

## RESULTS

### Long-term Tat expression led to poor short- and long-term spatial memory in iTat mice

To determine effects of long-term Tat expression on spatial memory, iTat mice and wild-type control mice (Wt) were fed with Dox-containing diet for 12 months and subjected to Morris water maze test (Fig. 1A), which consisted of 2-day pre-training sessions, nine training sessions and 5 probe tests for short-term spatial memory, and a 7-day break, one probe test for long-term memory [71]. Spatial memory was assessed by the percentage of the time spent in the target quadrant (Time at target quadrant %), path length travelled to the target quadrant (Distance to target quadrant), the percentage of time spent at the platform (Time at platform %), path length travelled to the platform (Distance to platform), the number of platform entries (Platform entries), and latency to platform. After nine sessions training, all mice had good ability to find the platform, with showing more time and distance in target quadrant and platform site, shorter latency and more frequent entries to platform location (data not show). Also, these intensive training cycles offered a chance to differentiate the short-term memory between Wt and iTat mice (probe test in day 13). Compared to Wt male mice, iTat male mice showed significantly less Time at target quadrant (Fig. 1B-I) [F(1,43)=7.675,* p*=0.008] and shorter Distance to target quadrant (Fig. 1B-II) [F(1,43)=4.944,* p*=0.031]. There were no differences in Time at target quadrant [F(1,43)=0.285,* p*=0.596] and Distance [F(1,43)=0.486,* p*=0.489] to target quadrant between Wt female mice and iTat female mice. iTat mice (both male and female) showed significantly less Time at platform (Fig. 1B-III) [F(1,43)=5.869,* p*=0.020], shorter Distance to platform (Fig. 1B-IV) [F(1,43)=6.674,* p*=0.013], and fewer Platform entries (Fig. 1B-V) [F(1,43)=6.947,* p*=0.012] than Wt mice (both male and female). In all these three measures, both male and female iTat mice exhibited a lower trend than male and female Wt mice. No difference in Speed was found between iTat mice (male and female) and Wt mice (male and female) (Fig. 1B-VI) [F(1,43)=0.368,* p*=0.547]. These results indicate that long-term Tat expression led to poorer short-term memory, particularly in male mice.

For long-term spatial memory, iTat male mice showed significantly less Time in target quadrant than Wt male mice [F(1,43)=4.159,* p*=0.048], and iTat female mice showed a less but not significantly Time in target quadrant than Wt female mice (Fig. 1C-I) [F(1,43)=1.229,* p*=0.274], which gave rise to a significant difference in Time in target quadrant between iTat mice (both male and female) and Wt mice (both male and female) [F(1,43)=5.023, *p*=0.030]. In addition, sex difference was also evident in both Wt and iTat mice as male mice exhibited more Time in target quadrant [F(1,43)=7.273, *p*=0.010]. In both male and female mice, iTat mice only showed shorter Distance in target quadrant (Fig. 1C-II) [F(1,43)=3.867, *p*=0.056], less Time in platform site (Fig. 1C-III) [F(1,43)=1.001, *p*=0.323], shorter Distance in platform site (Fig. 1C-IV) [F(1,43)=0.320, *p*=0.574], and fewer Platform entries (Fig. 1C-V) [F(1,43)=0.198, *p*=0.658]. But there was no difference in Speed between iTat and Wt mice (Fig. 1C-VI) [F(1,43)=1.512, *p*=0.226]. Moreover, there were significantly fewer iTat mice to reach to the platform site within 30 seconds than Wt mice in day 13 and 18 probe tests (Table. 1) (X^2^=5.232,* p*=0.022). These results further confirmed that long-term Tat expression negatively affected both short and long-term spatial memory.

**Figure 3.**
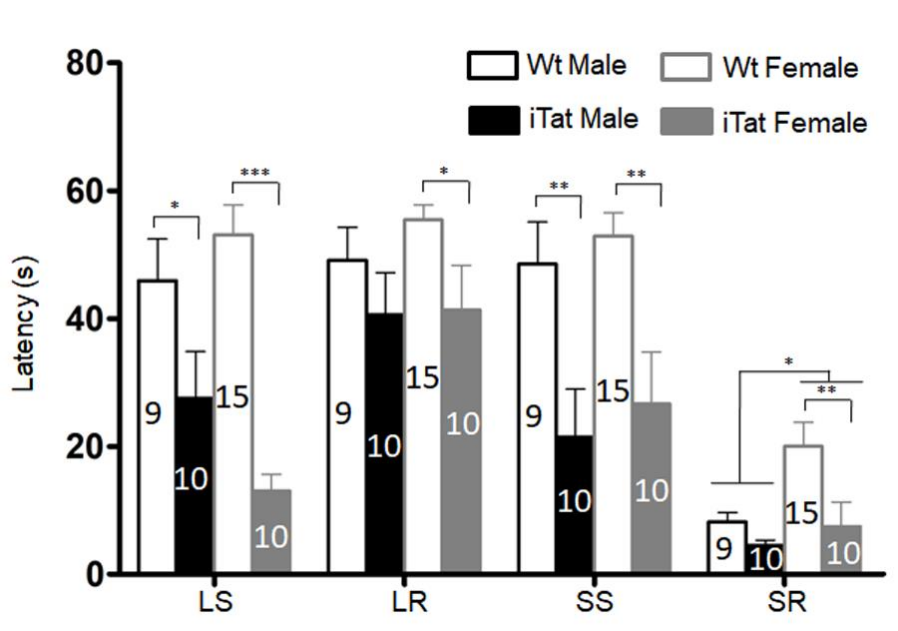
**Motor coordination and balance ability of iTat mice by bridge walk test**. The mice were placed into different types of raised beams, and the latency to fall from the beam was determined. The task was carried out with increasing difficulties and in the order of large square (LS), large round (LR), small square (SS), and small round (SR). The number of mice in each group was shown in the bar.

**Figure 4.**
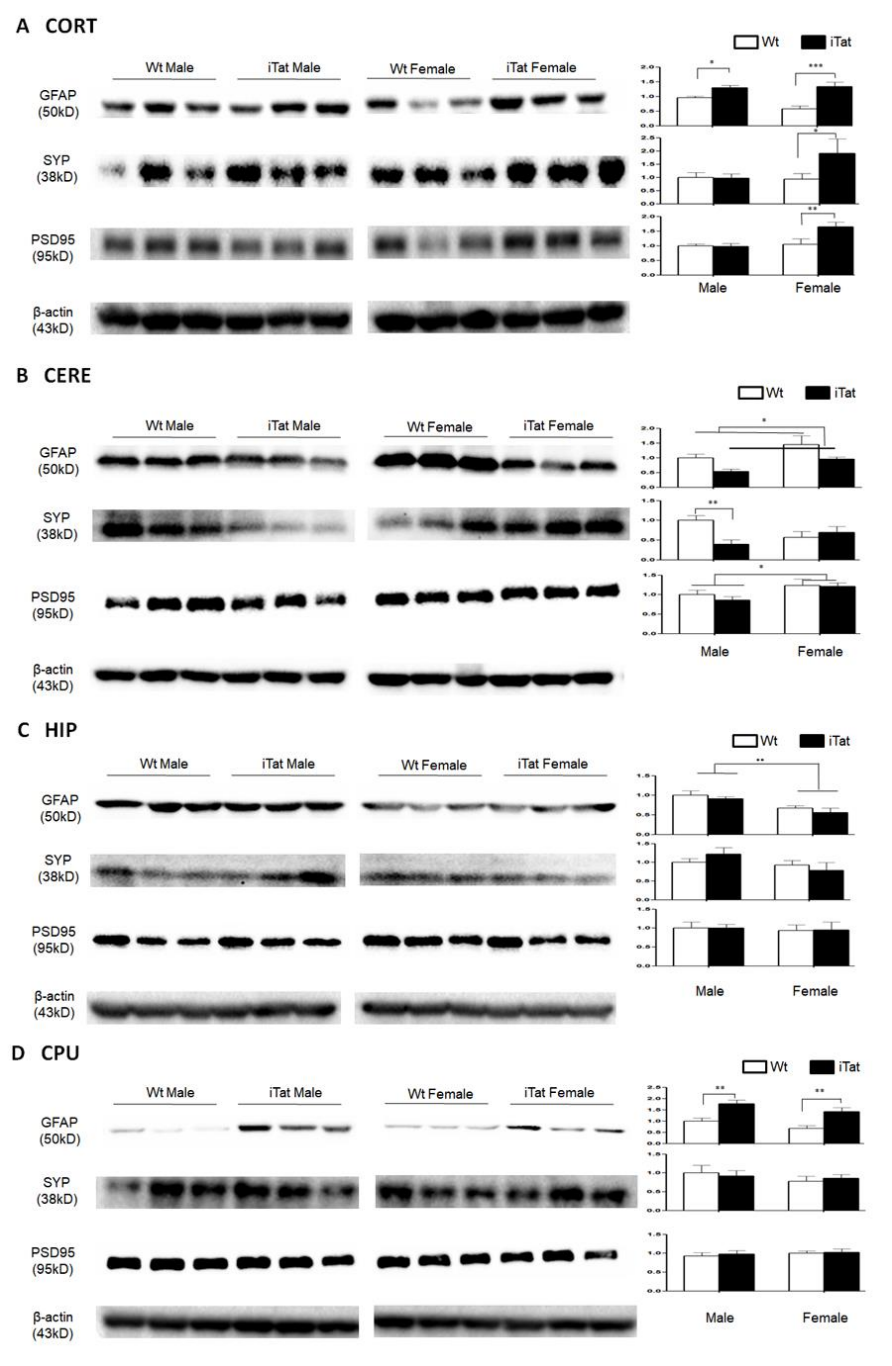
**Expression of GFAP, SYP and PSD95 in the brain of iTat mice**. Cortex (CORT, A), cerebellum (CERE, B), hippocampus (HIP, C) and caudate putamen (CPU, D) of the mice were dissected, homogenized for lysates, and analyzed for expression of GFAP, SYP and PSD95, by Western blotting. β-actin was used as an equal loading control. Six mice in each group were used for the analysis and three of them were randomly selected from the same SDS-PAGE for presentation. Protein expression in each group was normalized by Wt males and the relative level was shown at the right of the respective blots.

### Long-term Tat expression led to lower locomotor activity and impaired motor balance and coordination in iTat mice

The same mice were then subjected to open field test for the locomotor activity, which was assessed by the path length travelled (Total Distance) and the travel speed (Speed). For both male and female mice, iTat exhibited a shorter, although not significantly, Total Distance than Wt (Fig. 2A) [F(1,43)=1.535, *p*=0.222] and a lower, although not significantly, Speed than Wt (Fig. 2B) [F(1,43)=1.498, *p*=0.228]. The third behavioral test for these mice was bridge walk, in which latency to fall (Latency) was used to measure the motor balance and coordination. For both male and female mice, iTat showed worse performance in all four tasks LS [F(1,40)=27.581, *p*<0.001], LR [F(1,40)=4.809, *p*=0.034], SS [F(1,40)=17.668, *p*<0.001] and SR [F(1,40)=6.091, *p*=0.018] than WT (Fig. 3). Specifically, iTat female mice performed significantly worse in all four tasks LS [F(1,40)=29.392, *p*<0.001], LR [F(1,40)=4.232, *p*=0.046], SS [F(1,40)=9.724, *p*=0.003] and SR [F(1,40)=8.224, *p*=0.007] than Wt female mice, while iTat male mice performed significantly worse than Wt male mice in two task LR [F(1,40)=4.877, *p*=0.033] and SS [F(1,40)=8.134, *p*=0.007] (Fig. 3). These results suggest that long-term Tat expression impaired locomotor activity and motor balance and coordination, particularly more on female mice. In addition, there is a significant difference between male and female mice in task SR [F(1,40)=5.069, *p*=0.030], which indicates female mice have a better motor balance and coordination.

**Table 1:** Latency to platform within 30s.

Probe test Day	Genotype	Sex	Success	Failure	Total	*p*
13/18	iTat	Male	5	5	20	0.022*
		Female	6	4		
	Wt	Male	9	3	27	
		Female	14	1		

In probe tests on day 13 and 18, the success frequencies for reaching the platform within 30s in every mouse group were used to assess memory difference (day 13 for short-term memory and day 18 for long-term memory).

* :iTat total vs Wt total, <0.05. Pearson chi-square test was performed for Latency to platform in the water maze.

### Differential effects of long-term Tat expression on different brain regions

Acute and short-term HIV-1 Tat expression in iTat mice have been linked to astrocytes activation (astrocytosis), compromised neuronal integrity, and neurobehavioral deficits [57, 63, 72]. To determine the effects of long-term Tat expression on astrocytes activation and neuronal integrity, the brain of iTat mice was harvested at the end of the neurobehavioral studies, the cortex (CORT), striatum (caudate and putamen, CPU), hippocampus (HIP) and cerebellum (CERE) were dissected and analyzed for expression of glial fibrillary acidic protein (GFAP), a marker for astrocytes activation, synaptophysin (SYP), a pre-synaptic marker, and post-synaptic density protein 95 (PSD95), a post-synaptic marker. In CORT, GFAP [F(1,20)=26.908, *p*<0.001], SYP [F(1,20)=4.783, *p*=0.041] and PSD95 [F(1,20)=9.609, *p*=0.006] were all expressed at a significantly higher level in iTat female mice than Wt female mice (Fig. 4A). GFAP [F(1,20)=4.421, *p*=0.048] expression was significantly higher in iTat male mice than WT male mice, but SYP [F(1,20)=0.006, *p*=0.941] and PSD95 [F(1,20)=0.010, *p*=0.992] showed no differences between iTat male mice and Wt male mice. In CERE, GFAP expression was significantly lower in iTat male and female mice than Wt male and female mice (Fig. 4B) [F(1,20)=6.686, *p*=0.018], although iTat male mice [F(1,20)=4.286, *p*=0.052] or iTat female mice [F(1,20)=1.851, *p*=0.189] alone were lower, but not significantly, than Wt male mice or Wt female mice. SYP expression were significantly lower in iTat male mice than WT male mice [F(1,20)=10.035, *p*=0.005], but showed no differences between iTat female mice and WT female mice [F(1,20)=0.462, *p*=0.504]. PSD95 expression showed no differences between iTat mice and Wt mice [F(1,20)=0.432, *p*=0.518], but PSD95 expression was significantly higher in female mice than male mice [F(1,20)=5.885, *p*=0.025]. In HIP, GFAP expression was significantly lower in female mice than male mice (Fig. 4C) [F(1,20)=14.216, *p*=0.001]. There were no other differences in GFAP expression between iTat and Wt mice [F(1,20)=1.225, *p*=0.282]. There were also no differences in SYP [F(1,20)=0.067, *p*=0.789] and PSD95 [F(1,20)=0.002, *p*=0.968] expression between iTat mice and Wt mice, or between male mice and female mice [SYP: F(1,20)=1.387, *p*=0.253; PSD95: F(1,20)=0.129, *p*=0.723]. In CPU, GFAP expression was significantly higher in iTat male [F(1,20)=13.171, *p*=0.002] and female mice [F(1,20)=13.782, *p*=0.001] than Wt male and female mice (Fig. 4D). There were no differences in SYP [F(1,20)<0.001, *p*=0.991] and PSD95 [F(1,20)=0.191, *p*=0.667] expression between iTat mice and Wt mice, or between male mice and female mice [SYP: F(1,20)=0.850, *p*=0.368; PSD95: F(1,20)=0.492, *p*=0.491]. Taken together, these results provide the evidence for the first time that long-term Tat expression showed differential effects on different brain regions, which may contribute to specific behavioral changes of iTat mice.

**Figure 5.**
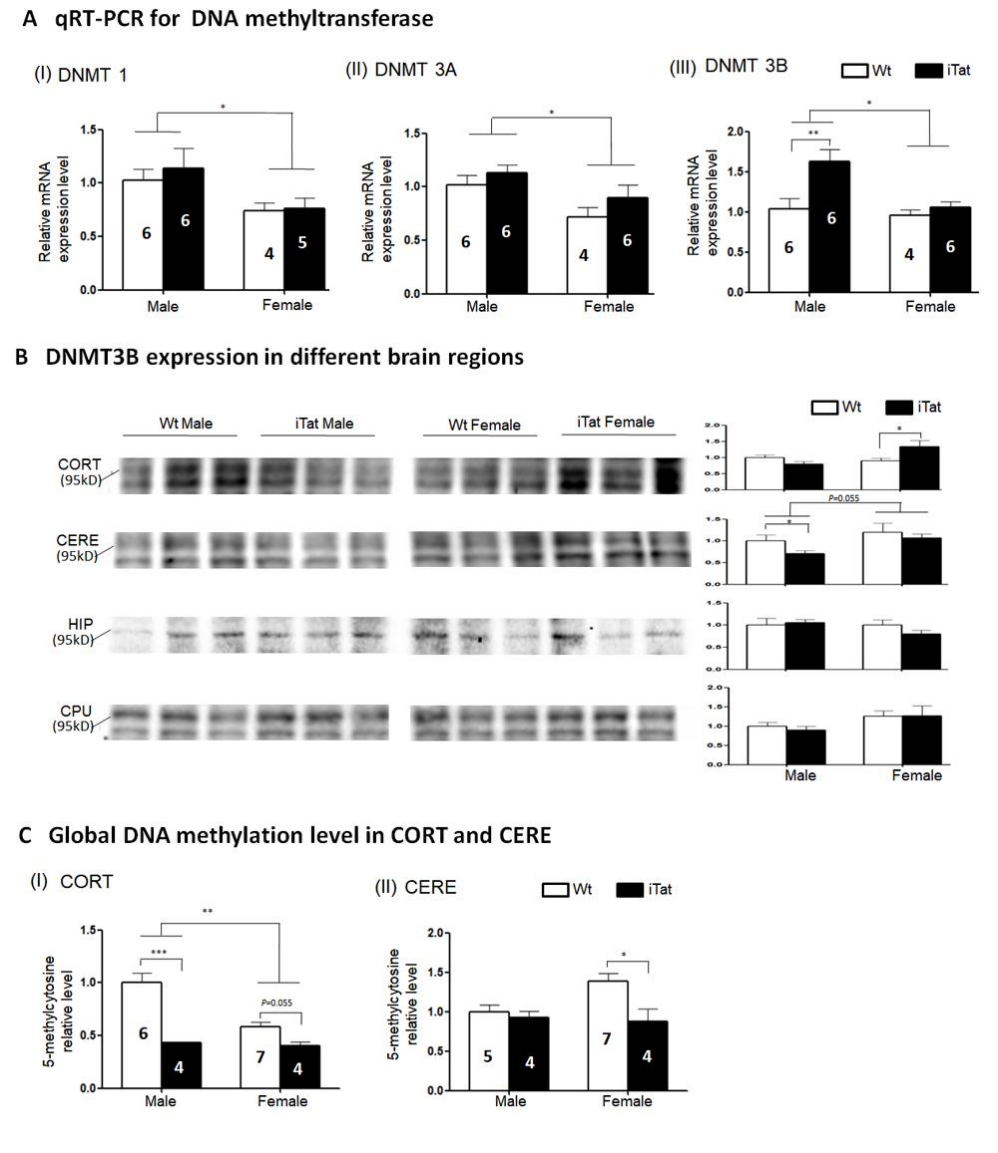
**DNA Methyltransferase expression and genomic DNA methylation in the brain of iTat mice**. qPT-PCR was used to screen the mRNA expression of DNMT1 (A-I) DNMT3A (A-II) and DNMT3B (A-III) in whole brain lysates, followed by Western blots to determine DNMT3B expression in different brain regions including CORT, CERE, HIP and CPU (two close bands were recognized by DNMT3B antibody in some brain regions) (B). Next, two brain regions, CORT and CERE, were selected to elucidate the genomic DNA methylation level by 5-methylcytosine ELISA. The number of mice was shown in the bar, except for Western blots where six mice were used in every group and three were randomly selected from the same SDS-PAGE for presentation. All data was normalized by Wt males and shown as a relative level. The internal control of Western blots in (B) was β-actin which was same to figure 4 in different brain regions.

**Table 2:** Summary of comparisons between different mice/sex and different brain regions.

		Male iTat vs Wt	Female iTat vs Wt	Total Male vs Female
Behavior	MWZ	↓↓	-	↑
	OPT	-	-	-
	BW	↓↓	↓↓↓↓	↓
	
CORT	GFAP	↑	↑	-
	SYP	-	↑	-
	PSD95	-	↑	-
	DNMT3B	-	↑	-
	5-MC	↓	-	↑
	
CERE	GFAP	-	-	↓
	SYP	↓	-	-
	PSD95	-	-	↓
	DNMT3B	↓	-	-
	5-MC	-	↓	-
	
HIP	GFAP	-	-	↑
	SYP	-	-	-
	PSD95	-	-	-
	DNMT3B	-	-	-
	
CPU	GFAP	↑	↑	-
	SYP	-	-	-
	PSD95	-	-	-
	DNMT3B	-	-	-

↑: significantly higher; ↓: significantly lower. The number of arrows indicates the number of indices in every behavioral test that have reached significant differences.

### Alterations of DNA methyltransferase expression and global genomic DNA methylation in the brain by long-term Tat expression

HIV infection or Tat expression alone has been shown to alter DNA methyltransferase (DNMT) expression [73, 74]. To determine effects of long-term Tat expression on DNMT expression in the brain, total RNA was isolated from the brain of iTat and Wt mice and analyzed for DNMT1, DNMT3A and DNMT3B mRNA expression using qRT-PCR. DNMT1 expression was significantly lower in female mice than male mice [F(1,17)=5.256, *p*=0.035], but showed no differences between iTat mice and Wt mice (Fig. 5A-I) [F(1,17)=0.381, *p*=0.545]. Similar results were obtained for DNMT3A expression (Fig. 5A-II) [male vs female: F(1,18)=8.328, *p*=0.010; iTat vs Wt: F(1,18)=2.563, *p*=0.127] and DNMT3B (Fig. 5A-III) [male vs female: F(1,18)=7.639, *p*=0.013], except for that DNMT3B was significantly higher in iTat male mice than Wt male mice [F(1,18)=8.657, *p*=0.009]. Thus, we then determined DNMT3B protein expression in each of the brain regions CORT, CERE, HIP and CPU. In CORT, DNMT3B protein was significantly higher in iTat female mice than Wt female mice [F(1,20)=6.831, *p*=0.017] but showed no difference between iTat male and Wt male mice (Fig. 5B) [F(1,20)=1.018, *p*=0.325]. In CERE, DNMT3B protein expression had higher trend in female mice [F(1,20)=4.170, *p*=0.055] than male mice and was significantly lower in iTat male mice than Wt male mice (Fig. 5B) [F(1,20)=5.248, *p*=0.033]. There were no differences in DNMT3B protein expression in HIP [F(1,20)=0.471, *p*=0.500] and CPU [F(1,20)=0.087, *p*=0.771] between iTat mice and WT mice or between male mice and female mice (Fig. 5B) [HIP: F(1,20)=1.311, *p*=0.266; CPU: [F(1,20)=3.764, *p*=0.067]. Lastly, we determined global genomic DNA methylation in CORT and CERE. Genomic DNA was isolated from CORT and CERE, the global genomic DNA methylation was analyzed for the percentage of methylated cytosine, i.e., 5-methylcytosine using ELISA. In CORT, 5-methylcytosine was significantly lower in iTat male [F(1,17)=38.754, *p*<0.001] and lower trend in female mice [F(1,17)=4.249, *p*=0.055] than Wt male and female mice and also significantly lower in female mice than male mice (Fig. 5C-I) [F(1,17)=12.115, *p*=0.003]. In CERE, 5-methylcytosine was significantly lower in iTat female mice than WT female mice [F(1,16)=10.944, *p*=0.004] but showed no differences between iTat male mice and WT male mice (Fig. 5C-II) [F(1,17)=2.072, *p*=0.169]. Collectively, these results indicate that long-term Tat expression altered DNA methyltransferase expression and genomic DNA methylation, which likely result in genomic remodeling, epigenetic changes, and changes of gene expression, and behaviors.

## DISCUSSION

In the study, we aimed to determine the relationship between long-term HIV Tat expression and neurobehavioral, pathological, and epigenetic changes in the brain using iTat mice. iTat mice were fed with Dox-containing diet for an extended period of 12 months and first analyzed for the behavioral changes. Long-term Tat expression led to poorer short-and long-term memory, lower locomotor activity and impaired coordination and balance ability (Fig. 1-3, Table 2). The effects of long-term Tat expression on memory were more pronounced in male mice and on coordination and balance ability were more pronounced in female mice (Fig. 1, Table 2). Then, astrocytes activation and neuronal integrity were assessed in the brain of those mice. Different neuroanatomical regions showed differential effects of long-term Tat expression on astrocyte activation and neuronal integrity. Long-term Tat expression resulted in more astrocytes activation and more loss of neuronal integrity in CORT and CERE, more astrocytes activation in CPU, and little effects in HIP (Fig. 4, Table 2). Lastly, DNMT expression and genomic DNA methylation were analyzed. DNMT1, DNMT3A and DNMT3B mRNA expression was significantly more in male mice than female mice (Fig. 5A). DNMT3B mRNA was significantly higher in iTat male mice than Wt male mice, while DNMT3B protein in CORT was significantly higher in iTat female mice than Wt female mice and DNMT3B protein in CERE was significantly higher in female mice than male mice (Fig. 5B). Tat-induced changes in DNMT3B protein expression appeared to be consistent with the changes in synaptic markers, specifically in CORT and CERE (Fig. 4). Meanwhile, a significantly lower level of 5-methylcytosine, an indicator of genomic DNA methylation was found in CORT in iTat male mice than Wt male mice and in CERE in iTat female mice than Wt female mice, while a lower level of 5-methylcytosine was found in CORT in iTat female mice than Wt female mice (Fig. 5C). All the differences may account for the differential effects of long-term Tat expression on behaviors between male and female mice, as CORT is important for learning and memory process while CERE is important for coordination and balance.

Aging is associated with physical, physiological and behavioral changes. In the study, we employed Morris water maze, open field and bridge walking to assess changes in learning, memory and motor function resulting from long-term HIV-1 Tat expression in the brain. Long-term HIV-1 Tat expression in the brain led to impaired short- and long-term memory but with more impairment’s male mice (Fig. 1, Table 1 and 2). Across species studies show that males perform better in spatial memory test than females in human [75] and that the sex difference in spatial memory is more pronounced in older mice [76], which implicates that males’ spatial memory is more sensitively suffered by HIV-1 Tat. Moreover, aging-associated menopause, accompanied by estrogen decline may be related to the worse memory in all females [77, 78], which may further fade the impact of HIV-1 tat on female mice. Our studies found that female mice appeared to travel longer distance in open chamber and stayed more time in bridge beam than male mice, and that female iTat mice performed worse than male iTat mice in bridge walking test (Fig. 2 & 3, Table 2). One of the confounding factors for the behavioral studies is the body weight, as body weight likely affects balance and coordination ability [79, 80]. Specifically, there is a negative correlation between body weight and balance and coordination ability at same age, and less body weight indicates better performance. We found that female mice weighed less than male mice at the end of 12 months of the Dox-diet (data not shown), meanwhile, females performed better in bridge task (Fig. 3), which suggests with the higher level of balance and coordination performance female mice are vulnerable to HIV-1 Tat-induced damage. Similar differences have been noted in HIV transgenic rat and Tat transgenic mice [67, 81], which may be due to selective neuronal vulnerability to HIV Tat [82].

Aging-related increases in astrocyte activation, determined by GFAP mRNA and protein expression, have been found in several areas of the brain including CORT [83, 84], CERE [83], striatum [85], and hippocampus [86]. We showed in this study that long-term Tat expression led to increased GFAP expression in both CORT and CPU, decreased GFAP expression in CERE and no changes in HIP (Fig. 4). In addition, we showed that GFAP expression in HIP of male mice was higher than that in HIP of female mice. As a sign of astrogliosis, GFAP is upregulated by aging, but downregulation also can be found entorhinal cortex [86]. Actually, astrocytes are highly heterogeneous with different phenotypes [87, 88], which may play different roles in aging process or the impact by HIV-1 Tat, further contributing to diversified results in different brain regions. SYP and PSD95, which modulate the synaptic plasticity and are involved in memory formation, have also been studied in aging process but their exact relationship to the aging process remain inconclusive [89-91]. We showed in present study that long-term Tat expression had no effects on SYP and PSD95 expression in HIP and CPU but differential effects in CORT and CERE depending on the sex (Fig. 4). A three-month Tat induction study has also shown no clear effects of Tat on HIP in a similar mouse model [92]. Treatment of recombinant Tat protein and short-term (21 days) Tat expression have led to decreased PSD95 and SYP expression *in vitro* [59, 93] and in CORT [94]. The findings from the current studies clearly show that Tat-induced neurotoxicity is brain region- and sex-dependent and suggest that long-term Tat expression could result in neuropathological changes similarly to aging-related changes in terms of GFAP, SYP and PSD95 expression in the brain.

Changes in DNMT3B expression have been detected in different cells and tissues during the aging process [95-98] as well as in B cells of HIV-infected individuals [74]. Our studies demonstrated that long-term Tat expression only led to increased DNMT3B mRNA expression in male mice (Fig. 5A). We further found that long-term Tat expression resulted in significant differences in DNMT3B protein expression in CORT of female mice (Fig. 5B). Interestingly, there appeared to be a paralleled change between SYP, PSD95, or both and DNMT3B (Table 2), which supports the hypothesis that the Tat-DNMT3B axis may be directly involved in accelerated aging process. Although DNMT1 and DNMT3A also have been shown associated with aging process and HIV positive people [73, 74, 95, 98], we only found the sex difference which not influenced by Tat. This discrepancy may due to species, tissue difference and more other factors involvement (such as HIV-1 gp120, Rev, and Nef proteins). We next showed that long-term Tat expression led to decreases in genomic DNA methylation in CORT of both male and female mice and in CERE of female mice (Fig. 5C). Genomic DNA hypomethylation has been linked to the aging process [99-101]. It is also of important note that a higher level of global DNA methylation has been detected in HIV-infected individuals [102], SIV-infected Rhesus macaques [103], and in Tat-treated microglia [103]. Sex differences in DNA methylation have been well documented [104-107]. It is clear that there are other DNMTs and other mechanisms such as demethylation involved in genomic DNA methylation during the Tat-induced accelerated aging process. Also, the dysregulation of global DNA methylation may further influence some aging related gene expression to accelerate aging process.

In conclusion, in this study we demonstrated that long-term Tat expression in the brain led to poorer memory and motor functions, and brain region- and sex-dependent dysregulation of neuropathological marker expression, DNMT3B expression, and genomic DNA methylation. HIV infection has been shown to accelerate biological aging process of HIV-infected individuals by five years in blood cells [108] and seven years in the brain [109]. The findings from our current study, along with the finding about the presence of Tat protein in the HIV-infected individuals under cART [12] raises the possibility that HIV-1 Tat contributes, at least in part, to accelerated aging process in HIV-infected individuals.
